# High Sensitivity and Ultra‐Broad‐Range NH_3_ Sensor Arrays by Precise Control of Step Defects on The Surface of Cl_2_‐Ndi Single Crystals

**DOI:** 10.1002/advs.202308036

**Published:** 2024-02-02

**Authors:** Bin Lu, Matthias Stolte, Dong Liu, Xiaojing Zhang, Lihui Zhao, Liehao Tian, C. Daniel Frisbie, Frank Würthner, Xutang Tao, Tao He

**Affiliations:** ^1^ State Key Laboratory of Crystal Materials and Institute of Crystal Materials Shandong University Jinan 250100 China; ^2^ Universität Würzburg Institut für Organische Chemie & Center for Nanosystems Chemistry Am Hubland 97074 Würzburg Germany; ^3^ Department of Chemical Engineering and Materials Science University of Minnesota Minneapolis Minnesota 55455 USA

**Keywords:** Kelvin probe force microscopy, NH_3_ sensor, organic single crystal, step defects, ultra‐broad detection range

## Abstract

Vapor sensors with both high sensitivity and broad detection range are technically challenging yet highly desirable for widespread chemical sensing applications in diverse environments. Generally, an increased surface‐to‐volume ratio can effectively enhance the sensitivity to low concentrations, but often with the trade‐off of a constrained sensing range. Here, an approach is demonstrated for NH_3_ sensor arrays with an unprecedentedly broad sensing range by introducing controllable steps on the surface of an n‐type single crystal. Step edges, serving as adsorption sites with electron‐deficient properties, are well‐defined, discrete, and electronically active. NH_3_ molecules selectively adsorb at the step edges and nearly eliminate known trap‐like character, which is demonstrated by surface potential imaging. Consequently, the strategy can significantly boost the sensitivity of two‐terminal NH_3_ resistance sensors on thin crystals with a few steps while simultaneously enhancing the tolerance on thick crystals with dense steps. Incorporation of these crystals into parallel sensor arrays results in ppb–to–% level detection range and a convenient linear relation between sheet conductance and semi‐log NH_3_ concentration, allowing for the precise localization of vapor leakage. In general, the results suggest new opportunities for defect engineering of organic semiconductor crystal surfaces for purposeful vapor or chemical sensing.

## Graphical abstract

With the advantage of unique step edge reactive sites and their controllability, a highly sensitive and ppb–to–% ultra‐broad‐range parallel NH_3_ sensor array is demonstrated and applied for quantitatively tracking NH_3_ leaks. The crucial significance of crystal steps and sensing mechanisms are established by KPFM. As a general strategy, the method will be expanded to other semiconducting single crystals.

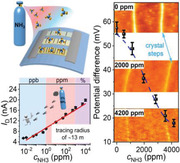

## 1 Introduction

Recently, flexible and wearable organic vapor sensors have attracted considerable attention owing to their portable applications in real‐time pollution monitoring in urban areas, tracing gas leaks during production, and analyzing the composition of exhaled air in medical diagnosis.^[^ ^1^ ^]^ Sensors that possess a low limit of detection (LOD), high selectivity, excellent response stability, and a wide detection range simultaneously are in high demand to effectively handle varying concentrations of vapor analytes in varied scenarios.^[^ ^2^ ^]^ Nevertheless, developing such sensors is still a technical challenge.

Generally, many properties of materials are structurally sensitive.^[^ ^3^ ^]^ A common approach to achieving low LOD and high‐sensitivity vapor sensors is the introduction of abundant adsorption sites by increasing the specific surface area of the adsorbing medium, such as porous,^[^ ^4^ ^]^ nanoparticle,^[^ ^5^ ^]^ network,^[^ ^5^, ^6^ ^]^ and dendritic array structures.^[^ ^7^ ^]^ Correlation of adsorption with an electrical property, such as resistance, that is easy to measure and to integrate with conventional microelectronics is highly desirable.^[^ ^8^ ^]^ Yet many high surface area adsorbents are not electrically conductive, or their conductivity is hard to tune consistently, or it degrades due to poor control of defect densities and trap‐state energies, leading to inevitable side effects on sensing performance reproducibility and operational stability.^[^ ^4^, ^9^ ^]^ These factors collectively prevent the practical implementation of real‐time and long‐term quantitative monitoring, e.g. accurate detection of vapor leakage sources. Meanwhile, the various surface features and localized energy states in the polycrystalline thin film also pose challenges in establishing a correlation between the number of effective adsorption sites and sensing performance, as well as in exploring precise sensing mechanisms. Therefore, there is a clear motivation for the deliberate engineering of resistive vapor sensors based on distinct and structurally well‐defined adsorption sites for vapor molecules. The step defects on the surface of the crystal seem to satisfy the aforementioned requirements. Our previous research has shown the presence of step edges with different potential variations on several organic single crystals.^[^ ^10^ ^]^ These crystal steps serve as traps for electrons/holes and impede charge transport as their density increases. Concurrently, defects at step edges exhibit a significant reactivity, allowing targeted surface doping for Cl_2_‐NDI and PDIF‐CN_2_ single crystals. This, in turn, leads to a notable enhancement in electron transport and enables the visualization of electron atmospheres through Kelvin probe force microscopy (KPFM).^[^ ^11^ ^]^ Therefore, we propose that organic semiconductor single crystal surfaces are an intriguing option for vapor sensing, in light of their high degree of order, their controllable conductivity, and the possibility for leveraging specific crystal‐analyte interactions through molecular design and crystal engineering. Currently, there are only a few reports concerning vapor sensors based on organic single crystals featuring tunable intrinsic adsorption sites.^[^ ^12^ ^]^

Herein, we demonstrate a strategy for achieving highly sensitive and ultra‐broad detection range NH_3_ sensors by controlling crystal step densities on the surface of single crystals of a n‐type semiconductor, N, N’‐bis‐(heptafluorobutyl)−2,6‐dichloro‐1,4,5,8‐naphthalene tetracarboxylic diimide (Cl_2_‐NDI). The crystal step edges, in the absence of the peripheral fluoroalkyl chain, not only demonstrate significant adsorption capabilities but also provide unique landing points for NH_3_ molecules. Reactive adsorption of NH_3_ at the step edge sites results in stark increases in surface conductivity that are directly proportional to the concentration of NH_3_ vapor; this is the basis of our resistive sensor. The mechanism for the conductivity increase is a reduction in the electron trap density at the step edges, which we demonstrate using surface potential imaging of the crystals by KPFM before and after NH_3_ exposure. Essentially, the charge transfer complex is created at the step edges, facilitating electron transfer from NH_3_ to Cl_2_‐NDI molecules and subsequently filling the traps. With a clear understanding of the mechanism, we engineer the step edge density on Cl_2_‐NDI crystals by growth conditions to achieve resistive NH_3_ sensors with tunable detection ranges and low, 2 V operating voltages. Fabricating resistive sensors in parallel, each with different detection ranges, results in an ultra‐high detection range composite sensor spanning part per billion (ppb) to part per hundred (%) concentrations in air. We demonstrate the practical operation of these sensors for tracing NH_3_ leaks in the ambient with detection distances as large as 13 m. In addition to demonstrating a highly effective NH_3_ sensor, our results provide an unusually clear demonstration of the connection between chemical reactivity and electronic effects at well‐defined, discrete defects in organic semiconductors. Our findings also suggest that there are additional opportunities for defect engineering on organic crystal surfaces for a variety of chemical sensing applications.

## 2 Results and Discussion

**Figure 1:**
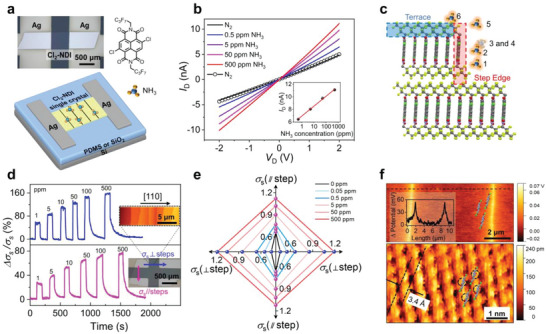
Crystal step edges act as reactive adsorption sites for the NH_3_ sensor. a) Device geometry based on a Cl_2_‐NDI single crystal; the molecular structure of Cl_2_‐NDI is also shown, and NH_3_ adsorption sites at the crystal step edges are illustrated. b) *I*_D_ *‐V*_D_ curves for a two‐terminal device at different NH_3_ concentrations. The inset displays *I*_D_ as a function of NH_3_ concentration. c) Schematic models of NH_3_ adsorbed at various positions on Cl_2_‐NDI molecular packing structure. d) The change in sheet conductance on exposure to NH_3_, which is sensitive to the orientation of steps with respect to the current direction. e) The evolution of *σ*_s_ anisotropy upon increasing *c*_NH3_. f) KPFM potential and high‐resolution AFM (HR‐AFM) images. Both panels show overlays of molecular packing. Note the difference in scale bars.

Figure 1a illustrates the device structure consisting of a lath‐like *β*‐phase^[^ ^10a^ ^]^ Cl_2_‐NDI single crystal with Ag electrodes (Figure S1, Supporting Information). The top‐contact geometry on the PDMS elastomeric substrate is utilized to ensure the charge transport layer and the reactive sites for analytes are co‐located on the upper surface of the crystal. This configuration most effectively reveals the relationship between the reactive adsorption sites, trap states, and electron transport. Crystal thickness dominated n‐type field‐effect conduction is demonstrated, attributed to the step edges with positive potential that serve as shallow traps and severely impede electron mobility and yield a small *V*_T_ shift (Figures S2 and S4, Supporting Information).^[^ ^10^, ^13^ ^]^ Upon exposure of crystals to the 500 ppm NH_3_ molecules, a notable increase in *σ*_s_ was demonstrated in thick crystals compared to slight changes in thin crystals, indicating surface steps not only serve as electron traps for charge transport but also are the principal reactive sites for electron transfer between Cl_2_‐NDI and NH_3_ molecules (Figures S2–S5, Supporting Information). Notably, the device conductivity is triggered by NH_3_ exposure even without gate voltage (*V*_G_ = 0 V), enabling gate‐free, two‐terminal resistance sensors (Figure 1b; Figure S3, Supporting Information). Consequently, the subsequent tests are conducted using the two‐terminal device configuration on the PDMS substrate unless otherwise specified. Furthermore, we observe a linear *σ*_s_–semi‐log *c*_NH3_ characteristic at a driving voltage of only 2 V. Such two‐terminal resistance sensors not only simplify the device structure but also can provide great opportunities for low power consumption and portability.

The investigation of anisotropic sensing performance was conducted on the (001) facet of a crystal to further validate that the observed reaction with NH_3_, as indicated by the *σ*_s_ increase, is indeed attributed to crystal step active sites rather than random structural defects, and also to explore the optimal orientation for sensing (Figure 1c). AFM topography imaging demonstrates step‐flow growth on Cl_2_‐NDI single crystals with the crystal long‐axis ([110] π‐stacking direction) perpendicular to the steps (Figure 1d).^[^ ^14^ ^]^ This orientation severely hinders charge transport in the [110] direction and results in a more pronounced fall in *σ*_s_ as step density increases, in comparison to charge transport that is parallel to the steps (Figure S6, Supporting Information).^[^ ^15^ ^]^ Thus, following exposure to NH_3_, *σ*_s_ exhibits a maximum increase of 160% perpendicular to the steps while only half as much increase occurs parallel to steps. This implies step edges play a crucial role as primary reactive adsorption sites. The picture is that NH_3_‐induced electrons erase a portion of the charge traps, allowing electron transport to be more dominated by the inherent packing structure of the molecules in the [110] direction and less influenced by step edge traps (Figure 1e). The results also confirm that the sensing performance is a channel phenomenon, rather than a contact effect.

Furthermore, the high‐resolution atomic force microscopy (HR‐AFM) image, Figure 1f, demonstrates the distances and an angle between the nearest neighboring molecules are 0.52 ± 0.02 nm, 0.62 ± 0.03 nm and 110° ± 2°, respectively, which align closely with the unit cell parameters (*a* = 5.1862(4) Å, *b* = 6.3422(5) Å, and *γ* = 109.6865(18)).^[^ ^10^ ^]^ This observation provides direct evidence that Cl_2_‐NDI molecules pack at step edges with the π‐conjugated core and chlorine atoms exposed to analytes, facilitating the adsorption of NH_3_, in contrast to the crystal terraces shielded by the fluorinated side chains (Figure 1c). This physical picture is further substantiated by an analysis of the total energy of NH_3_ absorbed on Cl_2_‐NDI molecules and the adsorption energy. The maximum adsorption energy (E_ad_) of ∼ −0.12 eV and lowest total energy (E_total_) of ∼ −345.85 eV are observed in Configurations 3 and 4 compared to the other adsorption sites, suggesting that the adsorption of NH_3_ at the lateral side of the NDI core is the most favorable (detailed seeing Figure S7 and Table S1, Supporting Information). Together, these findings provide unequivocal evidence that crystal steps act as the primary reactive adsorption sites. Moreover, crystal steps with homogeneous orientation and trap energy, particularly with tunable densities, offer possibilities for precise control over sensing performance.

**Figure 2:**
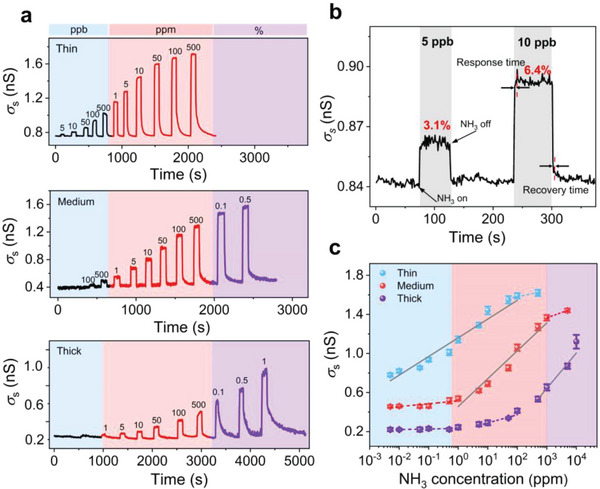
Crystal step density dependent behavior of two‐terminal NH_3_ sensors. a) Real‐time *σ*_s_ responses of crystals to varied *c*_NH3_. Crystal thicknesses are 1.2 µm (top), 2.5 µm (middle) and 4.2 µm (bottom), respectively. b) A partially enlarged panel of the thin Cl_2_‐NDI single crystal sensor in (a), showing the detailed shape of sensing curves and response/recovery time. The fast response/recovery times are 1 s and 2 s, respectively. The average response/recovery times are 1–5 s and 2–40 s in the linear range of *σ*_s_–*c*_NH3_ (5 ppb–50 ppm). c) Effective *c*_NH3_ detection (grey solid lines) for Cl_2_‐NDI single crystal sensors with various thicknesses. The blue, red, and purple colored regions represent ppb, ppm, and percent ranges of *c*_NH3_, respectively.

Initially our investigations focused on examining the relationship between crystal thicknesses (step densities) and sensing performance, as depicted in Figure 2a. The thin (1.2 µm) crystal sensor exhibits excellent sensitivity with LOD as low as 5 ppb, corresponding to an increase in *σ*_s_ of 3.1% (detailed in Figure 2b).^[^ ^16^ ^]^ Upon exposure to NH_3_ concentrations ranging from 5 ppb to 1 ppm, the sensor demonstrates rapid responses (1–2 s) and instantaneous recovery (2–15 s) after N_2_ flow over the crystal surface at a flux rate of 500 mL min^−1^, which surpasses most other electrical NH_3_ sensors.^[^ ^17^ ^]^ We argue that fast response is closely linked to the uniform alignment of steps and homogeneous trap energy that provide a consistent platform for electron trapping and release. This is evidenced by linear increases in *σ*_s_ with semi‐log *c*_NH3_ from 5 ppb to 50 ppm. As *c*_NH3_ further increases, *σ*_s_ levels off likely due to saturation of crystal step defect sites. By contrast, thick crystals demonstrate distinct differences in LOD and detection range. A much smaller initial *σ*_s_ value of only 0.2 nS (one‐quarter that of thin crystal) is present in the thick (4.2 µm) crystal sensor, which produces no measurable *σ*_s_ increase for *c*_NH3_ ≤ 1 ppm and then sluggishly responds for *c*_NH3_ between 1 ppm and 50 ppm. This behavior can be ascribed to a fraction of the number of relatively deep traps that occur at intersecting step edges on thick crystals and is reflected in a *V*_T_ shift (Figure S4, Supporting Information). A large number of isolated step edges and intersecting step edges on thick crystals means there is a spectrum of trap energies, shallow to deep, and this means that not only are there a large number of NH_3_ adsorption sites, but also that until step edge saturation occurs the rate of change with NH_3_ exposure will be more gradual than in thin crystals.^[^ ^8^, ^18^ ^]^ Stated another way, dense steps on thick crystals give rise to a superior capacity for accommodating a greater number of adsorbed NH_3_ molecules before achieving “ideal trap‐free” device performance. For thick crystals, *σ*_s_ undergoes a sharper increase when *c*_NH3_ > 50 ppm and does not reach saturation even at *c*_NH3_ = 1%.

Importantly, the sensing capabilities of medium‐thickness crystals lie in an intermediate range, which bridges the gap between thin (< 1.5 μm) and thick (> 3.0 μm) crystals (the definition of crystal thicknesses, see Figure S2, Supporting Information) and enables a comprehensive detection range by integrating sensors with various crystal thicknesses. This integration provides the possibility to realize a low LOD and high tolerance ppb–to–% wide‐range NH_3_ sensor for quantitative analysis.

**Figure 3:**
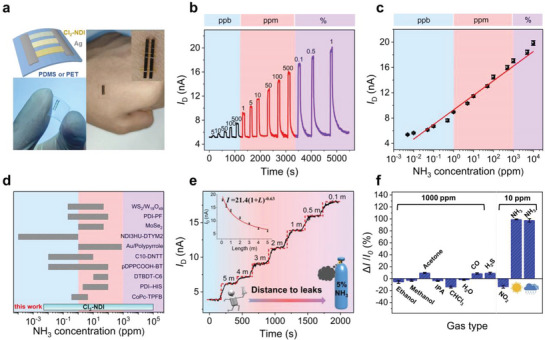
Sensing performance of parallel NH_3_ sensors. a) A parallel NH_3_ sensor on the flexible substrates PDMS or PET. Typically, at least three single crystals with different thicknesses are employed in a parallel configuration. b) Real‐time current responses of the parallel sensor array with exposure to 5 ppb–1% NH_3_. c) The current response in response to NH_3_ concentrations. d) A comparison of the detection range for Cl_2_‐NDI single crystal parallel sensor with other NH_3_ sensors (for details see Table S2, Supporting Information). e) The application for locating the source of NH_3_ gas leakage. The variation in current is recorded every meter away from leakage. The dwell time for each distance is 240 seconds. The inset depicts a nonlinear fitting (power‐law index) of *I*_D_ versus the leakage source distance (*L*). f) Tests for the selectivity and stability of parallel sensors. The sensor demonstrates clear specificity for NH_3_ when exposed to ten different analyte gases. The symbols of sun and rain cloud represent absolute dryness as measured in N_2_ and relative humidity of 75% in ambient, respectively.

To verify our hypothesis, we fabricated a parallel sensor array on the flexible substrate by using no less than three crystals with similar widths but varying thicknesses between 0.5‐6 μm (Figure 3a). Following the equation for parallel circuits ($I\;=\frac{U}{R}\;=\;\frac{U}{R_{\mathrm{thin}}}+\;\frac{U}{R_{\mathrm{med}}}+\frac{U}{R_{\mathrm{thick}}}$), the initial current is predominantly influenced by the thin single crystal with the lowest resistance. Therefore, when exposed to low *c*_NH3_ (ppb–level), the parallel sensor array is endowed with the features of thin single crystals. It exhibits a LOD as low as 5 ppb with a 3.8% increase in current and fast response/recovery characteristics (Figure 3b). As *c*_NH3_ increases, medium and thick crystals with more step edge reactive sites alternate in playing decisive roles in the response current, allowing for continuous detection (the detailed sensing performance before and after parallel is depicted in Figure S8, (Supporting Information). The upper sensing limit was not detected within the entire testing range from 5 ppb to 1% (Figure 3c). To the best of our knowledge, this is the first report of an NH_3_ sensor with such a broad detection range from ppb to % (Figure 3d and Table S2, Supporting Information). Importantly, the parallel sensor displays an outstanding linear response to *c*_NH3_ throughout the whole working range. The response current exhibits a 54% increase for each order of magnitude change in *c*_NH3_ and across a concentration range of six orders of magnitude. Such a high response resolution enables accurate calibration of *c*_NH3_ in combination with a shallow baseline drift (Figure 3b). In addition, the current baseline is only 5.2 nA at a bias voltage of 2 V, indicating an exceptionally low power consumption of ≈10 nW. The aforementioned merits, including two‐terminal device geometry on a flexible substrate, high sensitivity, ultrabroad detection range, a quick response time, and low power consumption, all indicate excellent potential for application in portable sensors for comprehensive and quantitative detection.

The detection of trace amounts of NH_3_ was conducted in a 20 m^2^ laboratory with an open door and window. A concentration of 5% NH_3_ gas was released half an hour before the test at a flow rate of 1 L min^−1^. As shown in Figure 3e, the parallel sensor exhibits a notable increase in current, ≈67%, when it is positioned at a distance of 5 meters from the source leakage point. The corresponding amplitude of the current increase lies within the *c*_NH3_ range of ≈500 ppb, which quantitatively agrees with results obtained from the utilization of commercial equipment. As the sensor approaches the leakage source, the response current shows a non‐linear increase, which may be caused by non‐incremental variations in *c*_NH3_ and uneven airflow rates within an open system. Following multiple tests, we determined that the maximum detection radius is ≈13 ± 0.4 m based on analysis of the current‐distance curve with a response current of 5% greater than the baseline (Figure 3e).

In addition to sensitivity and detection range, selectivity and air stability are also critical factors for practical sensor applications. ^[^ ^19^ ^]^ As shown in Figure 3f, the sensor shows a reduction in the current response when exposed to analytical gases with a pronounced polarity (‐OH), electron‐withdrawing capability (NO_2_), or destructive properties (CHCl_3_).^[^ ^9^, ^20^ ^]^ In contrast, the response current is significantly enhanced when exposed to reducing gases, such as NH_3_, CO, and H_2_S.^[^ ^1^, ^21^ ^]^ We observe that the response of our sensors to NH_3_ at 10 ppm is several times higher than ten other typical organic solvents and analyte gases at much higher concentrations, e.g. 1000 ppm, demonstrating a high level of selectivity (Figure 3f; Figure S13, Supporting Information). To explore air stability and reproducibility, especially the influence of humidity on the sensor, 75% humid air is substituted for dry N_2_ as the dilution and desorption gas. The parallel sensor array still maintains a high sensitivity with a signal reduction of only 3%, attributed to the hydrophobic fluoroalkyl chain on the crystal surface can effectively prevent the arbitrary invasion of O_2_/H_2_O (Figure 1c; Figures S9 and S10, Supporting Information). The results of the time‐dependent sensing performance indicate our sensor has excellent operational stability and reliability in realistic environments (Figures S9–S12, Supporting Information).

**Figure 4:**
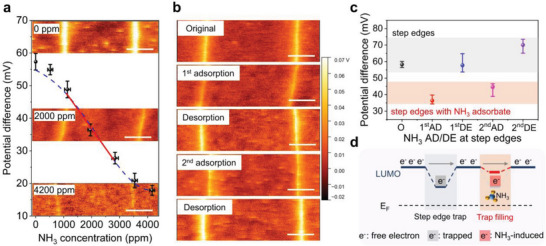
The sensing mechanism. a) KPFM step edge potential changes in a nearly identical region on a crystal surface under exposure to various NH_3_ concentrations in ambient air (75% relative humidity). b) KPFM potential maps of a crystal cyclic exposure to the same NH_3_ concentration. c) Corresponding potential differences for step edges with/without NH_3_ adsorption. d) The energy level diagram and electrons transport at step edges on the surface of Cl_2_‐NDI single crystals. The scale bars are 2 µm.

Understanding the sensing mechanism is the key to the optimization of materials and device configuration. To elucidate the microscopic origin of the interaction between Cl_2_‐NDI and NH_3_ as well as the reason for the response difference in the crystals with varying thicknesses, KPFM was carried out in ambient air at 75% relative humidity.^[^ ^22^ ^]^ The AFM topography images reveal Cl_2_‐NDI single crystals maintain a consistently smooth surface with an RMS of 0.1 nm and crystal step heights equivalent to single molecule lengths (≈1.8 nm) before and after NH_3_ exposure (Figure S14, Supporting Information). This suggests the introduction of NH_3_ molecules only results in surface adsorption on the Cl_2_‐NDI crystal and has no effect on the chemical bonding and packing structure. This result is supported by the XRD and XPS data, as depicted in Figures S15 and S16 (Supporting Information). However, the potential landscape demonstrates striking differences. The as‐grown crystal exhibits a potential difference of + 57 mV at step edges relative to the crystal flat terrace, which is consistent with the previous report measured in the glove box (Figure 4a).^[^ ^10^, ^11^ ^]^ When exposed to NH_3_, the step edge potential shows a gradual decrease upon the increasing of *c*_NH3_, and then nearly vanishes at *c*_NH3_ of 4200 ppm. This phenomenon is consistent with *σ*_s_ changes in sensing performance and suggests the step edge trap energy can be effectively modulated by varying *c*_NH3_. At the same time, the flat surface terrace does not show any potential change during the whole process by using Au as a reference (Figure S17, Supporting Information). This suggests the absence of charge transfer, which may be attributed to the hydrophobic nature of the surface and higher adsorption energy compared to step edges. These findings are visually represented in Figure S7 (Supporting Information) and quantitatively summarized in Table S1 (Supporting Information). Further investigation was conducted on cycle tests under the same *c*_NH3_. Here 2000 ppm NH_3_ was chosen because of the obvious decrease in step edge potential, and meanwhile, adsorbed NH_3_ molecules can be released in a few hours (Figures S18–S20, Supporting Information). Figures 4b,c demonstrate the potential alterations by switching NH_3_ and air analytes (detailed in Figures S21 and S22, Supporting Information). The potential drops from the initial +60 mV to +37 mV with NH_3_ exposure for 15 min. The decreasing potential implies that NH_3_‐induced electrons can passivate or erase partial traps, leading to an upward shift of the vacuum level and LUMO band edge (Figure 4d). The liberation of itinerant electrons facilitates an increase in conductivity. Subsequently, the step edge potential recovers to the original value of ≈ +60 mV after a few hours of storage in the humid environment, ascribed to the desorption of NH_3_. The reversible potential can be repeatedly achieved by alternating exposure to humid NH_3_ and air, which not only clarifies the sensing mechanism but also accounts for excellent operational stability. Consequently, the KPFM results provide visual proof that step edges are the only or primary reactive sites, and that the response to NH_3_ is a trap‐filling process. Electron spin resonance spectroscopy (EPR) data further corroborate amine can reduce Cl_2_‐NDI molecules in CHCl_3_ and [Cl_2_‐NDI]•^−^ radical anion is generated (Figure S23, Supporting Information).^[^ ^23^ ^]^ The process of electron transfer may also occur at crystal step edges.^[^ ^11^ ^]^

## 3 Conclusion

In conclusion, we have successfully devised a straightforward approach for achieving high sensitivity and ultra‐broad‐range (5 ppb to 1%) NH_3_ resistance sensor by introducing orientational crystal steps on the surface of n‐type Cl_2_‐NDI single crystals. The density‐tunable steps, which present unitary structural defects and trap states, serve multiple purposes. First and foremost, they provide uniform adsorption points for NH_3_ molecules and enable the formation of charge transfer complex, that contribute to high response speed, excellent selectivity, air stability and low operational voltage. Additionally, they offer a complementary detection range with the assistance of parallel sensors based on varying crystal thicknesses, resulting in a remarkable linear *I* – semi‐log *c*_NH3_ response in the entire testing range. As a consequence, the parallel sensor was capable of quantitative monitoring environmental *c*_NH3_ within a radius of 13 ± 0.4 meters, while also offering advantages such as low power consumption and portability.

Our study has also established the importance of crystal step edge traps in the NH_3_ sensing mechanism. KPFM results represent the first, intuitive description of crystal step edge potential changes with exposure to NH_3_/N_2_ (air) atmospheres, giving clear evidence for charge transfer between the Cl_2_‐NDI single crystal and NH_3_ molecules and shedding light on the sensing process. Our method, as a general strategy, will be applicable to a vast array of semiconducting single crystals with defect‐correlated surface potentials, including F_2_‐TCNQ and PDIF‐CN_2_.^[^ ^24^ ^]^ For example, our results suggest a new opportunity to reinvent organic single crystals at the molecular scale for purposeful vapor/chemical sensing by designing suitable functional groups exposed at step edges.

## 4 Experimental Section

### Crystal Growth

High‐quality *β*‐phase Cl_2_‐NDI single crystals were obtained by physical vapor phase transport (PVT).^[^ ^10^, ^11^ ^]^ The Cl_2_‐NDI powder placed at one end of the tube furnace was heated to 205 °C for sublimation, and then crystallized at the other end at 150 °C under a temperature gradient with N_2_ (10 mL min^−1^) as the carrier gas. Thin crystals (thickness < 1 µm) typically crystallize in 5–8 hours, while thick crystals (thickness > 3 µm) require 2–3 days. The thicknesses of crystals were measured by AFM (Bruker Dimension icon). As the thickness increases, there was a noticeable deepening of color from colorless (< 500 nm) to yellow (> 3 µm).

### Devices Fabrication

Polydimethylsiloxane (PDMS, Dow Corning Sylgard 184) coated Si or Polyethylene terephthalate (PET) substrates were prepared according to previous publications.^[^ ^25^ ^]^ Two terminal resistance sensor was fabricated by laminating lath‐like Cl_2_‐NDI single crystal onto a PDMS‐coated substrate, followed by 3 nm Cr/150 nm Ag electrode deposition by thermal evaporation under a vacuum of 1×10^−4^ Pa. The channel lengths of top‐contact device geometry are 600 µm and the widths vary according to the actual width of the crystals.

### Sensing Property Characterization

All the sensing experiments based on the Cl_2_‐NDI single crystals were carried out in a homemade Teflon test chamber with a volume of about 10 mL. The sample was wired to the test chamber using Cu wires (50 µm) and Ag paint. A Keithley 2612B source‐measure unit was used to apply the drain voltage and measure the resulting current under the various NH_3_ concentrations that were obtained by combining commercially available concentrations of 10 ppm, 1000 ppm, and 10% of NH_3_ with pure N_2_. Due to the restriction of the gas mixture system, a maximum of 2000‐fold dilution was allowed. Consequently, the NH_3_ sensor arrays can only test a minimum concentration of 5 ppb, despite the detection of hundreds of parts per trillion (ppt) level may be achievable according to the *I* – *c*_NH3_. The test chamber was connected to a solution of diluted sulfuric acid, which serves as a means of tail gas treatment. *σ*_s_ was extracted by the equation of $\sigma_{s}=\frac{L}{W}\;\;\times \frac{I}{V}$, where *L* and *W* are the channel length and width, respectively. The operation voltage was 2 V.

### Kelvin Probe Force Microscopy (KPFM)

Measurements were performed with Bruker Dimension icon mode AFM, equipped with a Bruker SCM‐PIT conductive probe (Pt/Ir, resonance frequency 60–90 kHZ, k = 1.2–5.5 N/m, R_C_ = 25 nm). All KPFM scans were performed in an open system, utilizing the lift mode. The lift height was set to 10 nm and a direct current (DC) bias voltage of 2000 mV was applied. To mitigate the desorption of NH_3_ at the crystal steps during KPFM measurements, the corresponding *c*_NH3_ directly flows through the surface of the crystal by using the syringe. The scan rate was set at 0.5 Hz to ensure that the entire image was completed in 15 minutes and to minimize the potential discrepancies between the measured and actual values.

### Electron Paramagnetic Resonance (EPR)

EPR tests were conducted by Bruker A300 electron paramagnetic resonance spectrometer at a low temperature of 100 K. The microwave power was 19.45 mW with a frequency of ≈9.65 GHz. A field modulation of 100 kHz was applied during the experiments. The concentration of Cl_2_‐NDI solution in CHCl_3_ was 10^−4^ mol L^−1^.

### X‐ray Diffraction (XRD) and X‐ray Photoelectron Spectra (XPS)

XRD spectra of Cl_2_‐NDI single crystals were recorded based on a Bruker D8 Advance X‐ray diffractometer with Cu Kα irradiation and *λ* = 1.54 Å. A continuous scan was used with a scan range of 3°–40°, step size set to 0.01° and a scan speed of 10°/min. XPS was carried out by a Thermo Scientific ESCALAB Xi+ XPS with an Al Kα X‐ray monochromatic source (1486.3 eV) at 10^−8^ Torr. The analyzer was set to a pass energy of 100 eV for survey scans with an energy step size of 1.0 eV, and 20 eV for high‐resolution scans with an energy step size of 0.05 eV.

### Density Functional Theory (DFT) Studies

DFT calculations were implemented in Vienna Ab‐inito Simulation Package (VASP), accounted by the projected wave (PAW) method with the Perdew‐BurkeErnzerhof (PBE) generalized gradient approximation (GGA).^[^ ^26^ ^]^ The energy cutoff for plane wave expansions was set to 500 eV, and the vacuum layer was set to 15 Å to avoid interaction between layers. The structural optimization was completed for energy and force convergence set to 1.0×10^−4^ eV and 0.02 eV Å^−1^, respectively. The Brillouin zone 3 × 3 × 1 meshes for structural optimization. Grimme's DFT‐D3 methodology was used to describe the dispersion interactions.^[^ ^27^ ^]^ Charge transfer was analyzed by the Bader charge analysis method. The adsorption energy (E_ad_) was defined as:

(1) $$E_{\mathrm{ad}}=\;E_{\mathrm{NDI}+\mathrm{NH}3}\;-E_{\mathrm{NDI}}-E_{\mathrm{NH}3}$$

where E_NDI + NH3_, E_NDI_ and E_NH3_ were the total energy of NDI with adsorbed NH_3_, pristine NDI, and NH_3_ molecules, respectively.

## Conflict of Interest

The authors declare no conflict of interest.

## Author Contributions

T.H. and X.T. conceived the initial idea and designed experiments. B.L. completed device fabrication, sensing property characterization, and KPFM measurements. D.L. and X.Z. helped with the device fabrication. L.Z. and L.T. performed the electronic conductivity measurement. F.W. and M.S. provided the highly purified Cl_2_‐NDI material and together with C.D.F. contributed to the scientific discussion of the results. B.L., X.T., and T.H. wrote the manuscript.

## Supporting information

Supporting Information

## Data Availability

The data that support the findings of this study are available from the corresponding author upon reasonable request.
