# Supplementary material for: High Sensitivity and Ultra‐Broad‐Range NH3 Sensor Arrays by Precise Control of Step Defects on The Surface of Cl2‐Ndi Single Crystals
Source: Adv Sci (Weinh). 2024 Feb 2;11(14):2308036. doi: 10.1002/advs.202308036 (PMC11005746; doi:10.1002/advs.202308036)
Supplement: Supplementary file 1 — Supporting Information [file ADVS-11-2308036-s001.pdf]

ADVANCED SCIENCE
Open Access

Supporting Information

for *Adv. Sci.*, DOI 10.1002/advs.202308036

High Sensitivity and Ultra-Broad-Range NH_3 Sensor Arrays by Precise Control of Step Defects on The Surface of Cl_2-Ndi Single Crystals

Bin Lu, Matthias Stolte, Dong Liu, Xiaojing Zhang, Lihui Zhao, Liehao Tian, C. Daniel Frisbie, Frank Würthner, Xutang Tao and Tao He**

Supporting Information

High Sensitivity and Ultra-broad-range NH_3 Sensor Arrays by Precise Control of Step Defects on the Surface of Cl_2-NDI Single Crystals

Bin Lu[1], Matthias Stolte[2], Dong Liu[1], Xiaojing Zhang[1], Lihui Zhao[1], Liehao Tian[1], C. Daniel Frisbie[3], Frank Würthner[2], Xutang Tao[1] and Tao He[1]**

B. Lu, D. Liu, X. Zhang, L. Zhao, L. Tian, Prof. X. Tao, Prof. T. He
State Key Laboratory of Crystal Materials and Institute of Crystal Materials, Shandong University, Jinan 250100, China
E-mail: txt@sdu.edu.cn; the@sdu.edu.cn

Dr. M. Stolte, Prof. F. Würthner
Universität Würzburg, Institut für Organische Chemie & Center for Nanosystems Chemistry, Am Hubland, 97074 Würzburg, Germany

Prof. C. D. Frisbie
Department of Chemical Engineering and Materials Science, University of Minnesota, Minneapolis, Minnesota 55455, USA

1. Calculation method

LOD calculation: The limit of detection (LOD) is calculated using the following equation from reference:[1]

LOD > 3 S.

S: standard deviation of response. At a certain concentration, if the response is more than three times the standard deviation, this concentration can be identified as the limit of detection of the sensor.

The standard deviation of response in our thin crystal sensor is <0.16% from the $\Delta\sigma_S/\sigma_S$ baseline, while the response at 5 ppb NH_3 concentration is 3.1%, extremely higher than the noise level. Therefore, we think the LOD of the thin crystal sensor is far less than 5 ppb. Nevertheless, due

to the limitations imposed by the experimental conditions, we were unable to test sensor performance at NH_3 concentrations of < 5ppb. The calculation of LOD for parallel sensors is also based on the same method.

Response time and recovery time: The response time is the time needed for the current change to reach 90% after the NH_3 supply is turned on, and the recovery time is the time for the current to decrease from 90% to initialization after the NH_3 is turned off.[2]

2. Cl_2-NDI single crystal sensors for NH_3 sensing

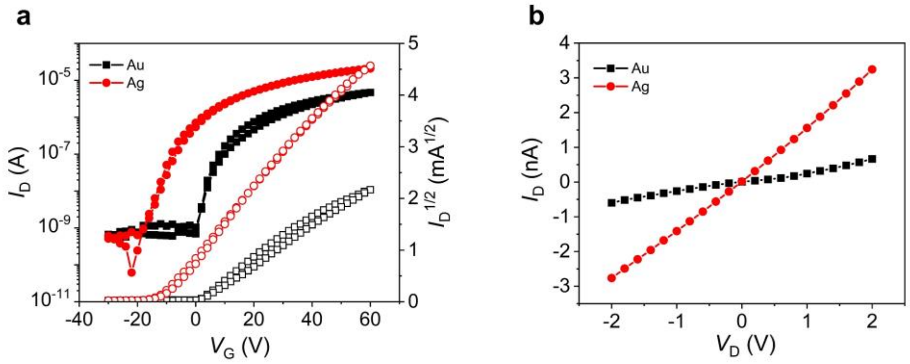

Figure S1. The comparison for Cl_2-NDI single crystal FETs with Au and Ag electrodes. a) Transfer characteristics. b) Output curves at V_G of 0 V. The device using Au electrodes demonstrates a significant decrease in I_D and an increase in V_T as compared to the device with Ag electrodes. This observation indicates a notable increase in injection resistance for Au electrodes and can be attributed to the considerable disparity between the work function of Au (–5.1 eV) and the LUMO energy level (–4.0 eV) of the n-type semiconductor Cl_2-NDI.

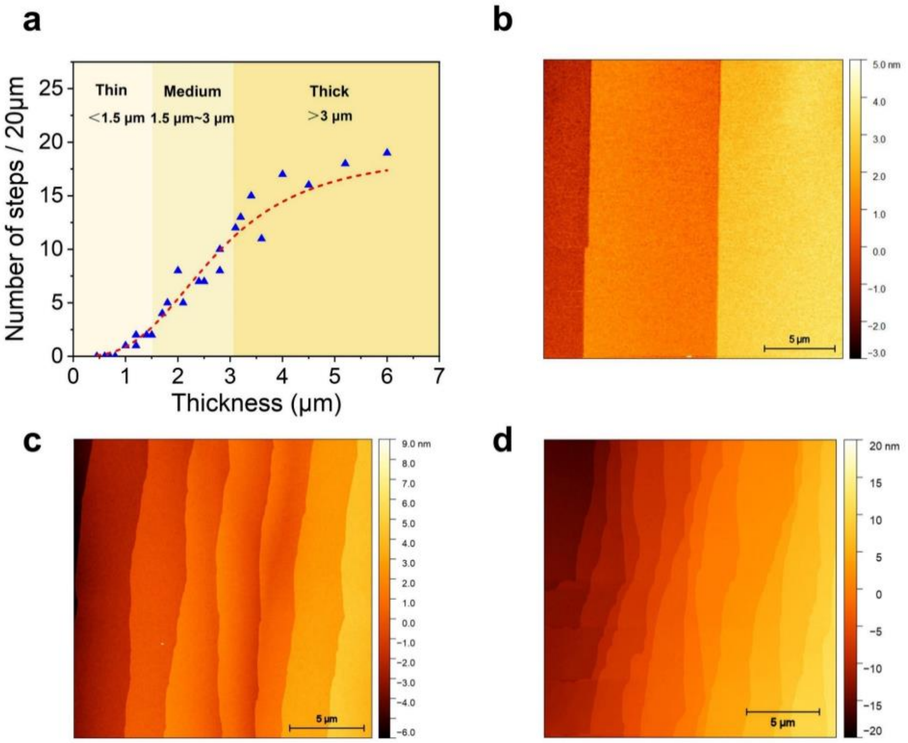

Figure S2. a) Step density on the surface of Cl_2-NDI single crystal versus crystal thicknesses. b–d) AFM images of Cl_2-NDI single crystals with thicknesses of (b) 1 μm, (c) 2.5 μm and (d) 4 μm, respectively. Crystal steps are vertical to crystal long-axis ([110] direction) and the step density exhibits an upward trend as the crystal thickness increases. Thin crystals are characterized by the presence of fewer than 3 steps within a 20 μm range along the crystal long-axis. The thicknesses of crystals are usually less than 1.5 μm. On the other hand, thick crystals are described as having more than 10 steps within a 20 μm range along the crystal long-axis. The thicknesses of crystals exceed 3 μm. As crystal thickness increases, the lateral area of step edges and surface-to-volume ratio are correspondingly enhanced. Since crystal step edges serve as reactive sites for NH_3 molecules, the sensing range is directly proportional to the step density and, by extension, the surface-to-volume ratio.

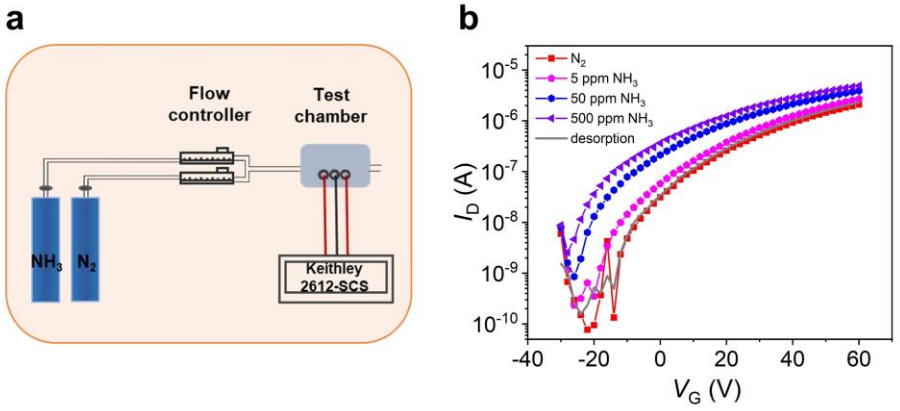

Figure S3. a) Schematic diagram of the sensor testing system. b) Transfer characteristic curves of Cl_2-NDI single crystal FETs based on Ag electrodes upon exposure to various NH_3 concentrations.

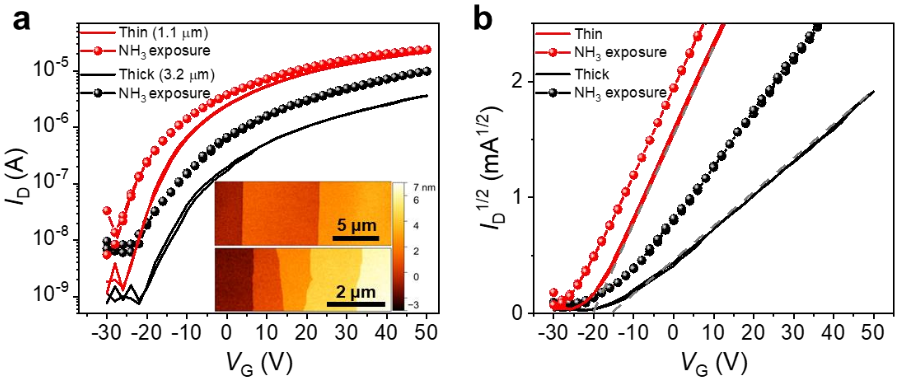

Figure S4. (a) I_D–V_G transfer characteristics for Cl_2-NDI single crystal FETs before and after NH_3 exposure. Inset to a): corresponding AFM topography images for the thin (top) and thick (bottom) single crystals, respectively, highlighting differences in step edge density. (b) $I_D^{1/2}$–V_G plot for Cl_2-NDI single crystal FETs before and after NH_3 exposure. Compared to the thin single crystal FET (red), the thick one (black) shows a notable decrease in I_D and a slight positive shift in V_T, which is ascribed to the presence of shallow traps with positive potential of +55 $\pm$ 5 mV (~2 k_BT/e) at step edges and a small number of deep traps at the intersection of crystal steps, respectively. With exposure to 500 ppm NH_3, although a significant increase in σ_s was demonstrated in thick crystals, it was still unable to reach the "idea" performance based on thin single crystals with negligible step edge traps. It is attributed to the charge transport primarily occurring at the interface between the crystal and SiO_2 dielectric layer, but the adsorption of NH_3 at

the upper surface of single crystals. It is noted that the device is entirely triggered at V_G= 0 V and shows performance gains with increasing NH_3 concentration. It eliminates a gate voltage of several tens of volts required to sustain the device in an "on state" to compensate for charge depletion caused by structural defects and gas exposure (see Table S2). This result enables the utilization of two terminal resistance sensors, simplifying the device structure while simultaneously ensuring that both the charge transport layer and the reactive sites of analytes are situated on the upper surface of the crystal.

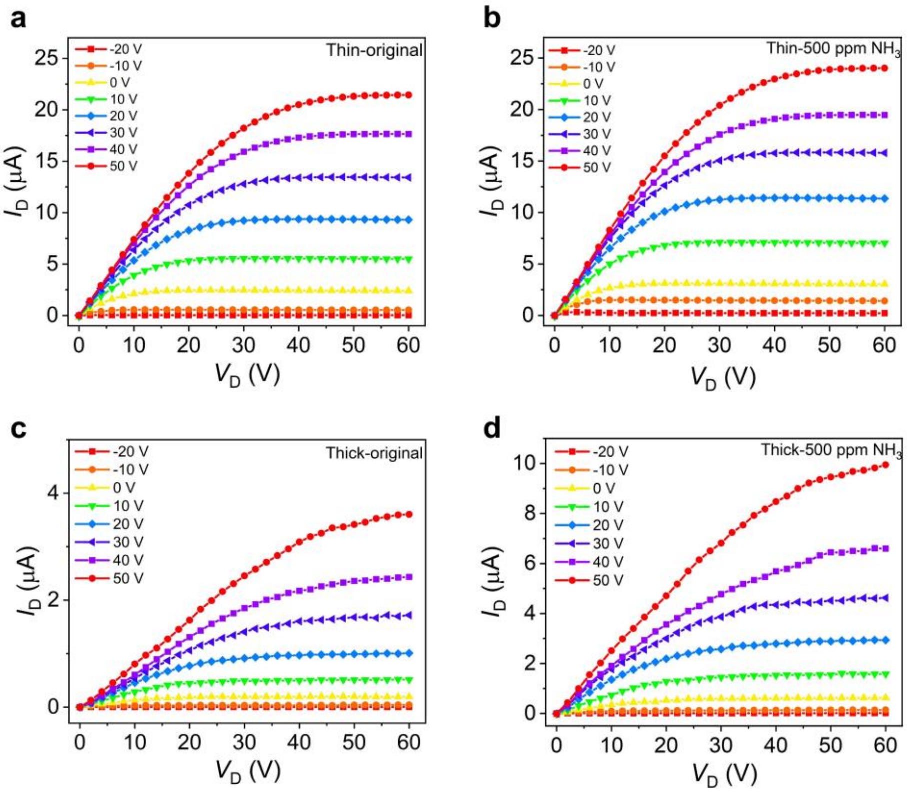

Figure S5. The comparison of output characteristic curves of sensors before and after exposure to 500 ppm NH_3. a, b) a thin (1.1 μm) and c, d) a thick (3.2 μm) Cl_2-NDI single crystal. The channel lengths of both devices are 600 μm, and the channel width of the thin crystal device and thick crystal device is 740 μm and 950 μm, respectively.

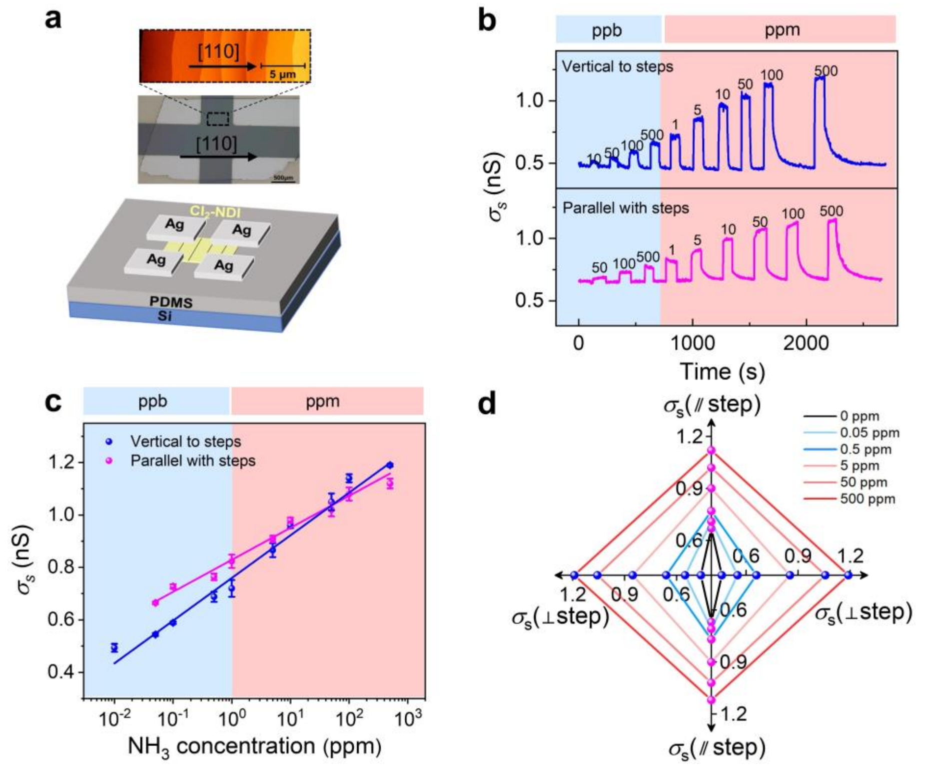

Figure S6. a) Cross-shaped two-terminal device geometry of Cl_2-NDI single crystal sensor. The optical micrograph and AFM topography image show that the steps are almost perpendicular to the crystal long-axis ([110] direction). b) The orientation of steps determined sensing performance. c) The variation of σ_S along the vertical and parallel steps as a function of NH_3 concentrations. d) Corresponding anisotropic sensor performance.

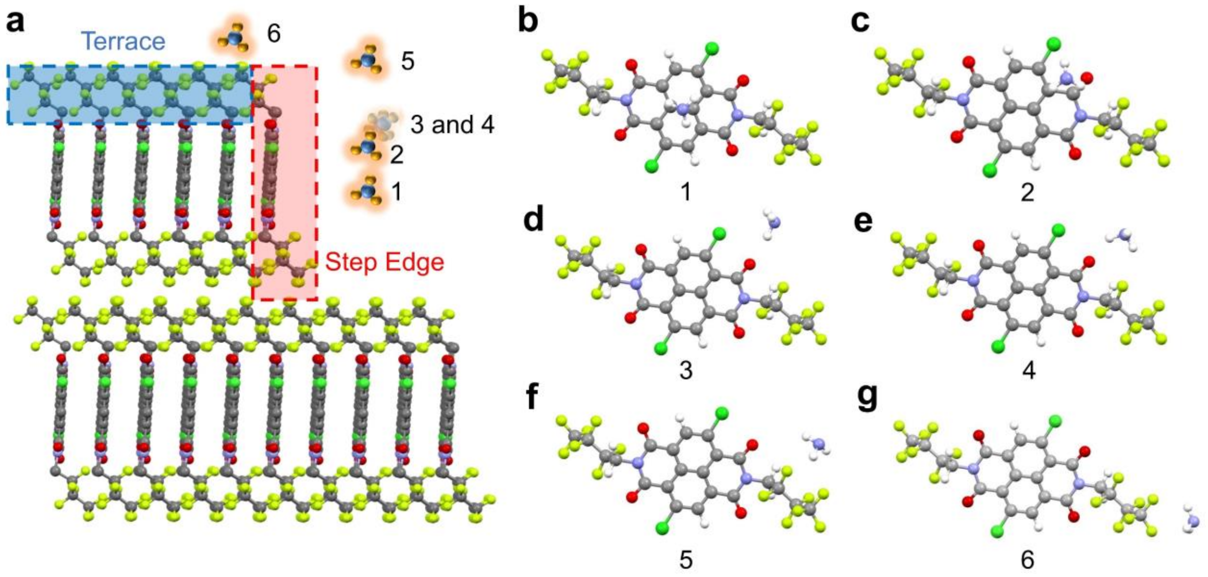

Figure S7. Schematic models of NH_3 adsorbed at various positions on Cl_2-NDI molecules (a) and partially enlarged details of these adsorption configurations (b–g). b,c) NH_3 adsorbed on the facet of the NDI core; d,e) at the lateral of the NDI core with two different orientations; f,g) in the middle and at the end of the fluoroalkyl chain.

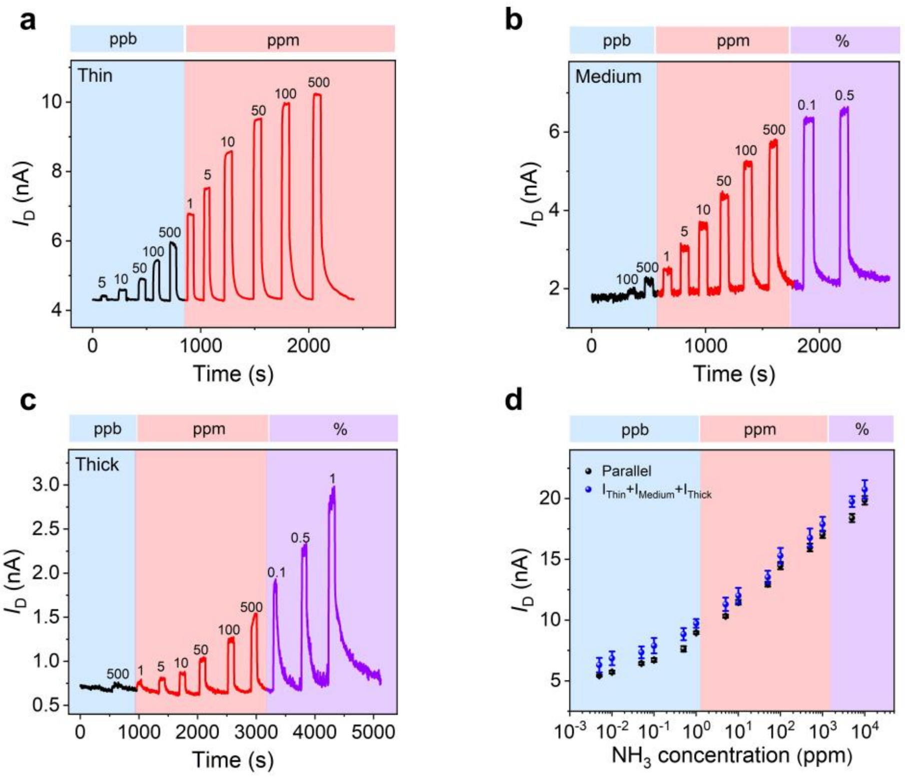

Figure S8. Sensing performance for three Cl_2-NDI single crystals with varying thicknesses before and after being connected in parallel. a–c) Real-time current measurements of three individual sensors. The thicknesses are 1 μm, 2.5 μm and 4 μm for thin, medium and thick single crystals. The limits of detection and response currents are 5 ppb (3.1%), 100 ppb (6.3%,), and 1 ppm (6.8%), respectively. d) The comparison of the response current upon c_{NH3} increases before and after parallel connection. The black dots represent the increase in response current for the genuine parallel device; The blue dots represent the sum of the response currents of the three individual sensors. The results of the comparison indicate that the parallel sensor possesses both the low detection limit of thin crystals and the tolerance of thick crystals.

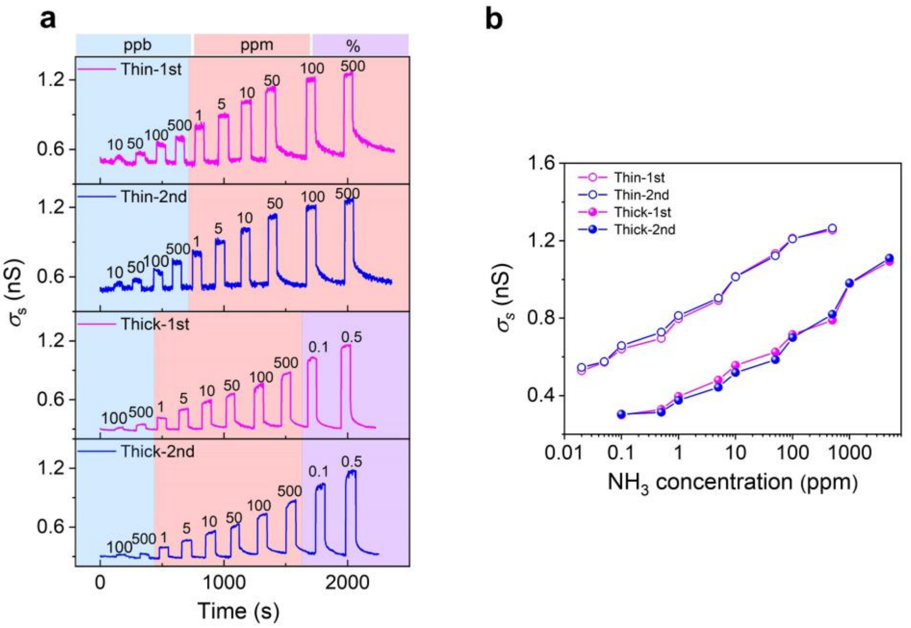

Figure S9. Cycle sensing experiments of Cl_2-NDI single crystals with different thicknesses. a) Real-time σ_s responses of a thin (1.3 μm) and a thick (3.5 μm) crystal exposed to varied c_{NH3} with N_2 as the diluent gas. b) The consistency of the σ_s response during cycle tests. A nearly identical change in σ_s curves is observed under twice exposures, exhibiting excellent reproducible sensing performances.

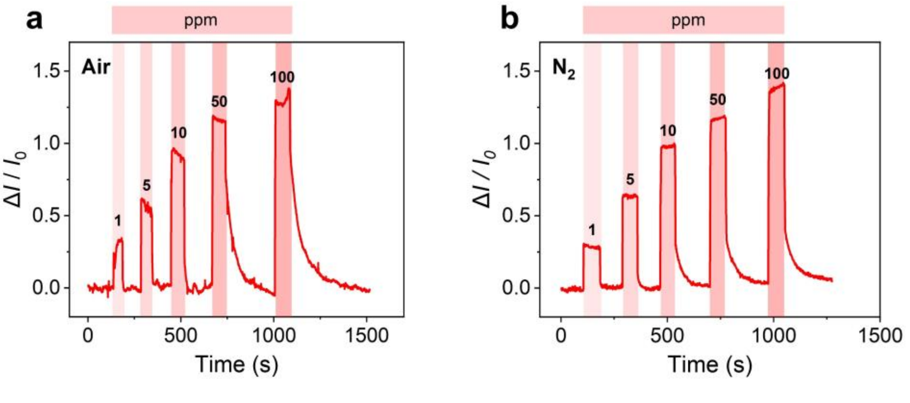

Figure S10. Sensing performance of Cl_2-NDI single crystal sensor exposed to NH_3 with 75% humid air (a) and dry N_2 (b) as the dilution gas, respectively. As we can see, there is only a small variation (3% at c_{NH3} = 100 ppm and the maximum deviation of 6.4% at c_{NH3} = 100 ppm) in the current response for sensor exposure to humid air (a) and dry N_2 (b), which indicates

excellent operational stability in an ambient environment. Additionally, the noise of the base-line current shown in (a) is caused by air pump vibration.

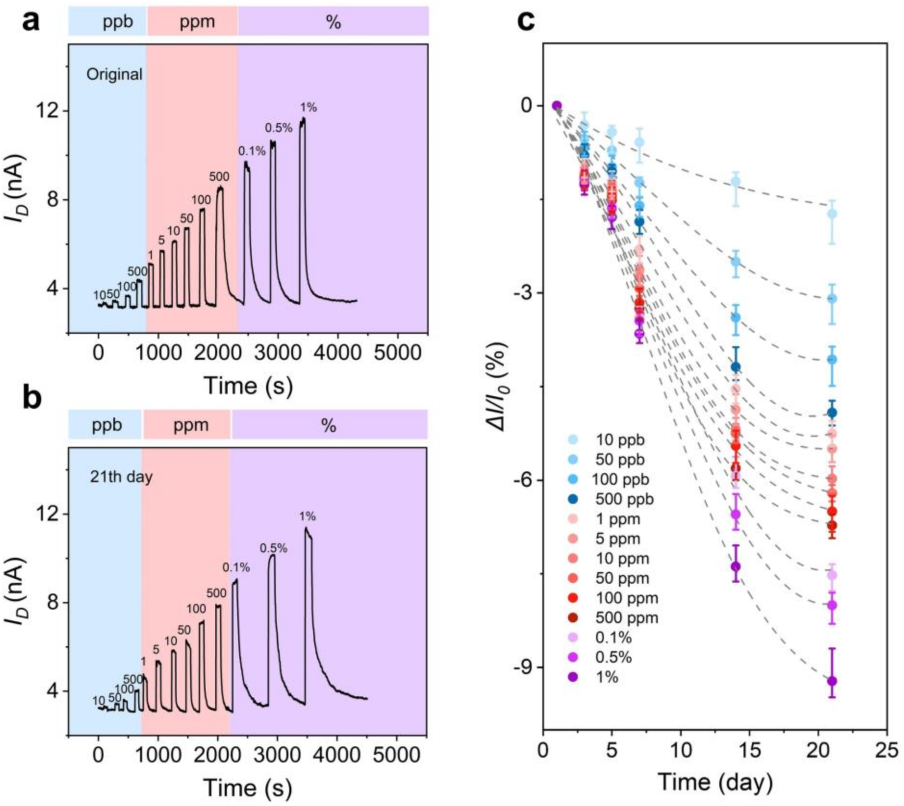

Figure S11. The stability of a parallel sensor array over three weeks. a,b) The sensing performance of a parallel sensor array on the first day (a) and stored in air for 21 days (b). c) Time-dependent stability for the sensor exposed to varied NH_3 concentrations. The response current decreases over time as a result of the influence of humidity and atmospheric oxygen. The sensor undergoes a maximal degradation of 9.2% at the relative humidity between 35% and 55% in the dry winter. The gradual deceleration of degradation indicates the absorption of H_2O and O_2 tends to saturation at the crystal step edges.

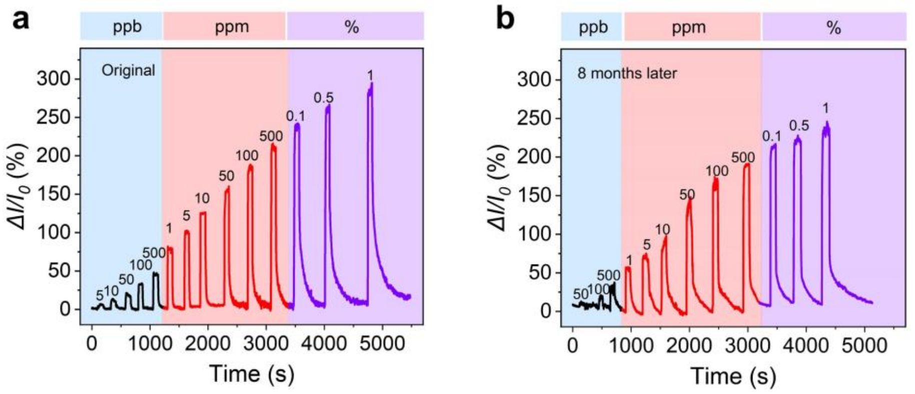

Figure S12. The long-term stability test for the parallel sensor array stored in air for eight months. Real-time current response of (a) the fresh sensor exposed to varied concentrations of NH_3 and (b) after storage in air for eight months later. The maximum and average degradation are 38% and 22%, respectively. These results indicate that our sensor possesses a comparatively high level of stability in the realm of organic gas sensors, which can be attributed to hydrophobic fluorinated side chains that shield crystal terraces from the environment. The humidity of the storage environment is between 75% in summer and 35% in winter.

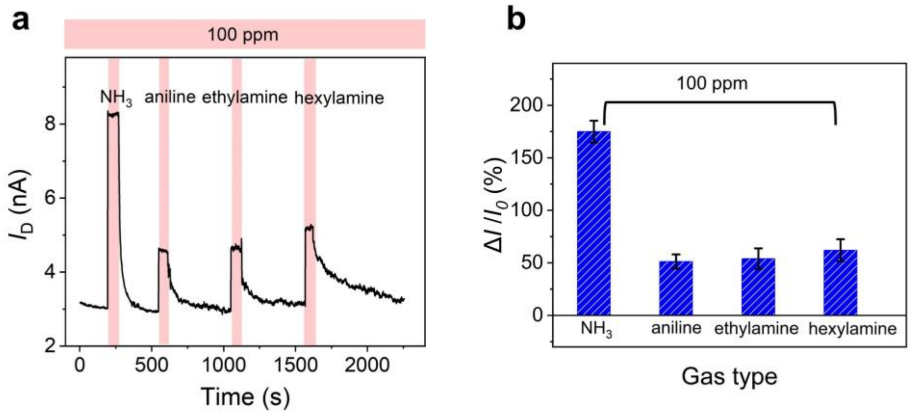

Figure S13. The selectivity of a Cl_2-NDI sensor for various types of orangic amines at the concentration of 100 ppm. The sensor exhibits significantly higher response with current of 180% increase compared to amiline (51%), ethylamine (53%) and hexylamine (61%). The Cl_2-NDI sensors have exceptional selectivity for organic amines. However, they are unable to

distinguish between different types of organic amines due to factors such as electron affinity, adsorption energy, steric hindrance and orientation of different amines at crystal step edges.[3]

3. Sensing mechanism

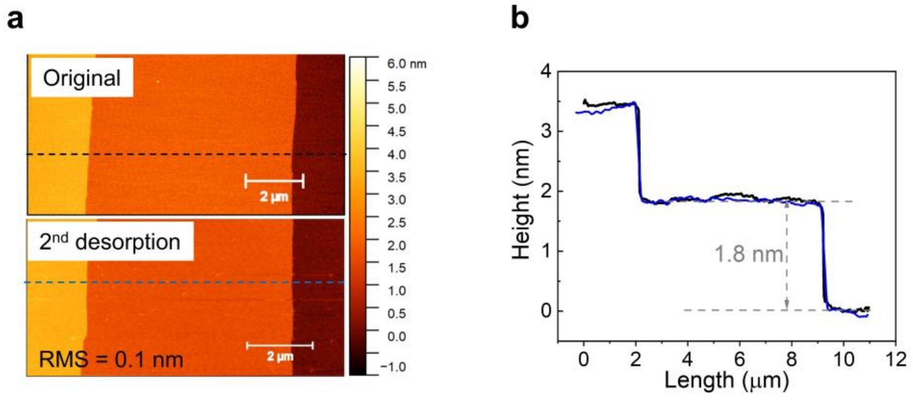

Figure S14. The crystal morphology changes upon repeated exposure to NH_3. a) AFM topography. b) Corresponding height profiles along the dashed line of Cl_2-NDI single crystal before and after cyclic exposure to 2000 ppm NH_3 atmosphere.

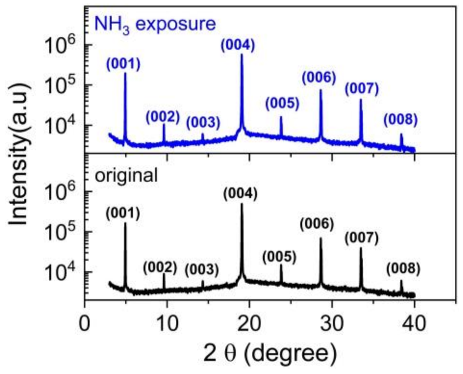

Figure S15. X-ray diffraction for a Cl_2-NDI crystal before and after exposure to NH_3. The original crystal XRD result shows multiple diffraction peaks, which corresponds to molecular layer spacing of 18.80 Å and is consistent with the *c*-axis parameters. After exposure to 2000 ppm NH_3 vapor, the intensity and position of the diffraction peaks remain unchanged, indicating that the introduction of NH_3 is only surface adsorption and has no effect on the chemical bonding and stacking arrangement of the Cl_2-NDI molecule.

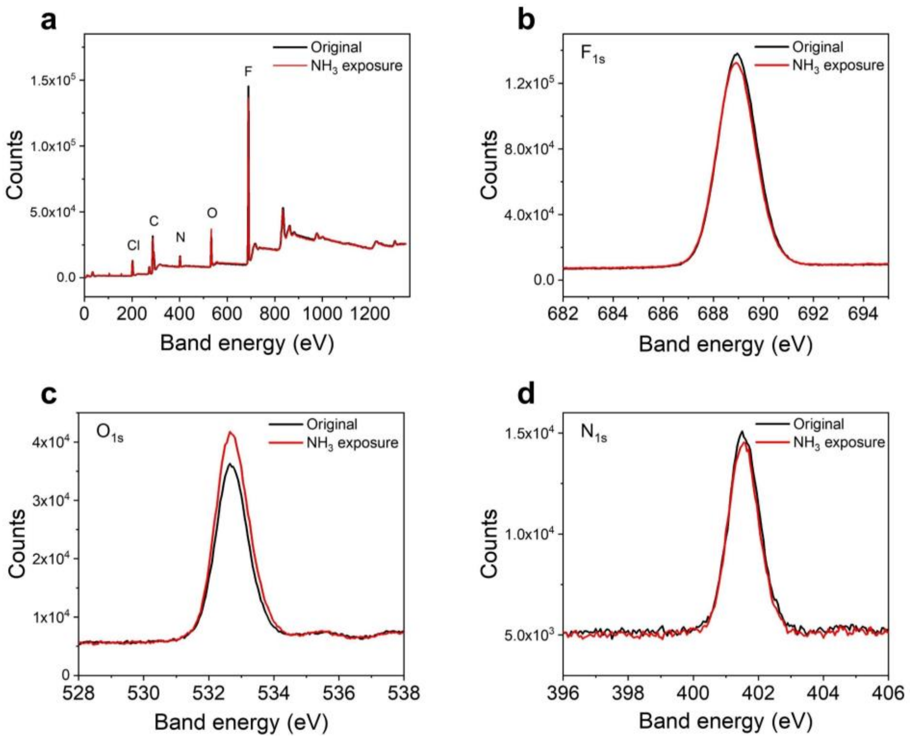

Figure S16. XPS profiling spectra for a 1.5 μm-thick Cl_2-NDI crystal before and after exposure to 2000 ppm humid NH_3 atmosphere. a) The complete XPS spectra of the Cl_2-NDI crystal. Compared with the original Cl_2-NDI crystal, only the O_{1s} peak exhibits a slightly elevated intensity and becomes wider after exposure to 2000 ppm humid NH_3, which may arise from a small amount of H_2O/O_2 molecules existing at step edges in the form of physical or chemisorption. b-d) High-resolution XPS F_{1s} (b), O_{1s} (c) and N_{1s} (d) element spectra. The signal of C=O locates at 532 eV in the O_{1s} spectra, and N–C=O from the imide group at 401 eV in the N_{1s} spectra, which both remain unchanged, suggesting the introduction of NH_3 molecules does not affect chemical bonding and packing structure.

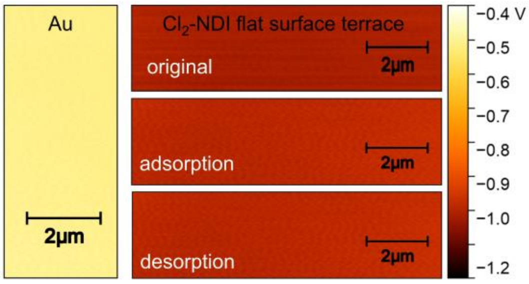

Figure S17. KPFM surface potential images of Cl_2-NDI single crystal flat surface terrace with exposure to 2000 ppm NH_3. Compared to Au as a reference, there is no potential difference for the flat surface before and after NH_3 exposure, suggesting crystal step edges are the only or primary reactive sites for charge transfer between NH_3 and Cl_2-NDI molecules.

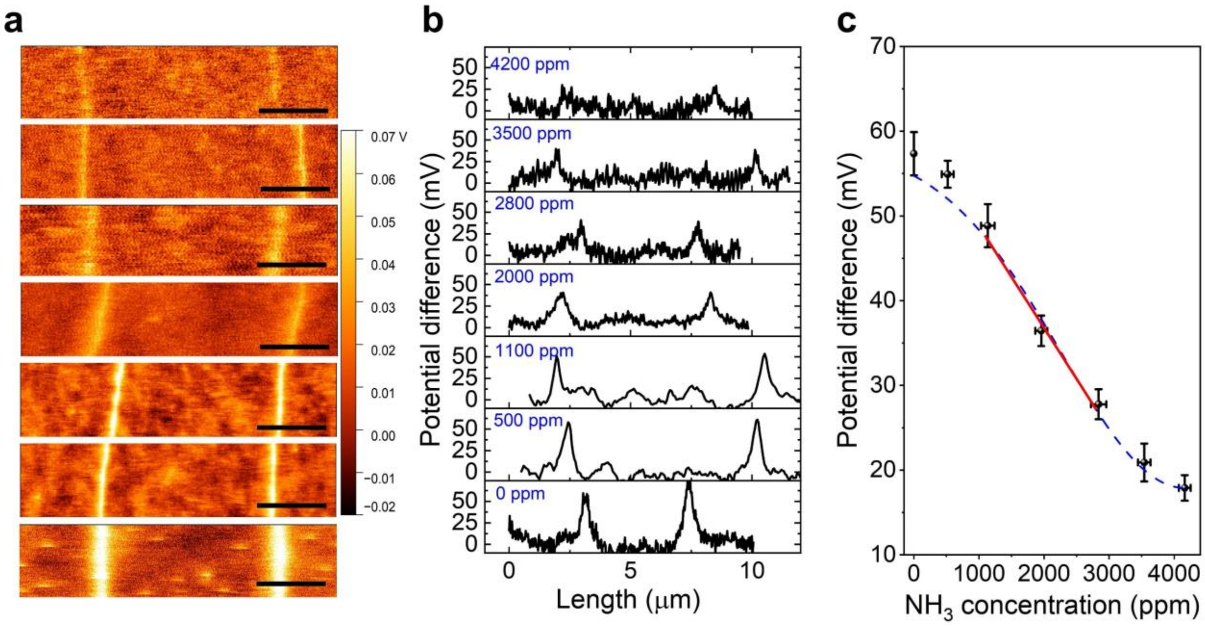

Figure S18. The surface potential of a Cl_2-NDI single crystal by exposed to varied concentrations of NH_3. a) KPFM surface potential images. b) Corresponding surface potential profiles. c) Corresponding step edge potentials as a function of NH_3 concentrations (blue). Note: Cl_2-NDI single crystal undergoes desorption prior to each instance of exposure to varying concentrations of NH_3. Scale bars, 2 μm.

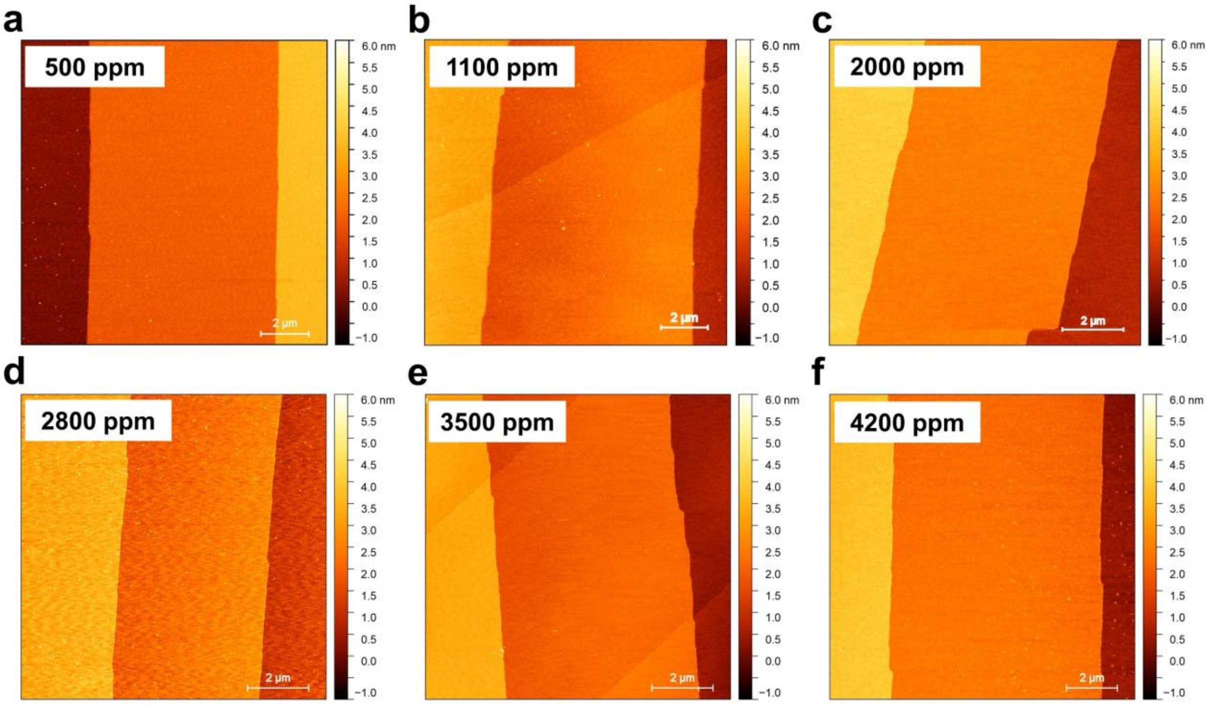

Figure S19. AFM topography images of Cl_2-NDI single crystal under varying concentrations of NH_3.

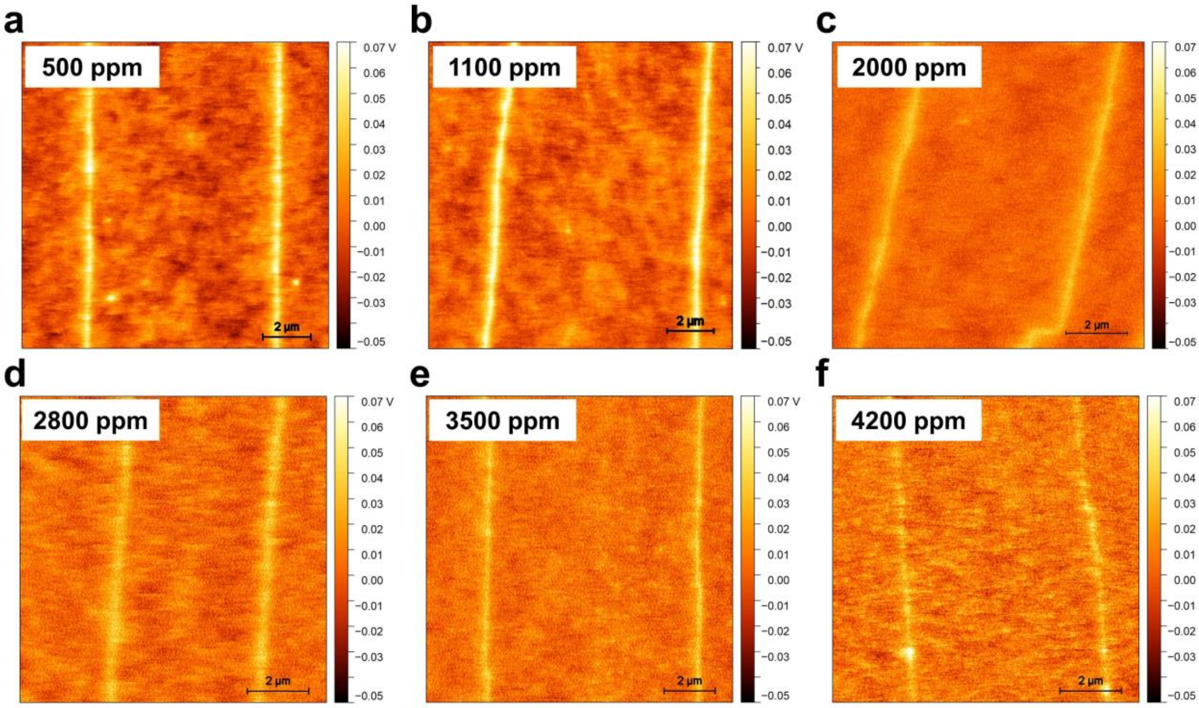

Figure S20. Corresponding KPFM surface potential images for Figure S19.

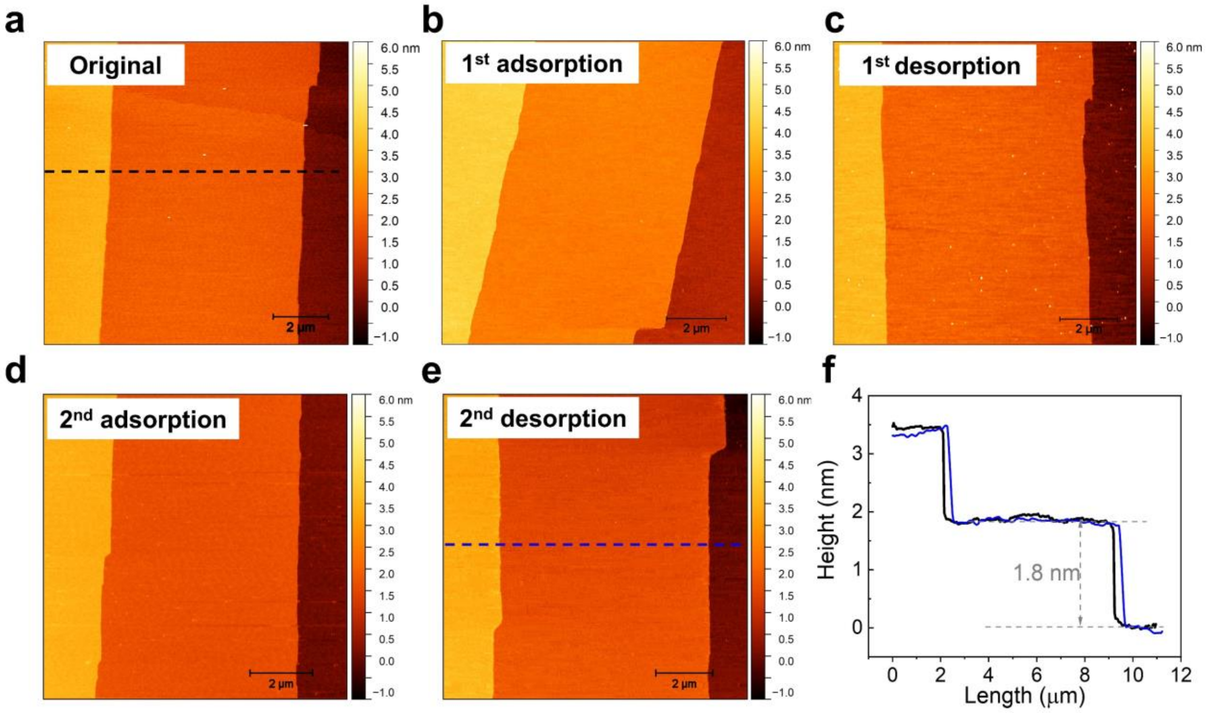

Figure S21. a–e) AFM topography of Cl_2-NDI single crystal by alternating exposure to 2000ppm NH_3 and humid air. f) The comparison of height profiles for the original crystal surface and that after two cycles of adsorption and desorption.

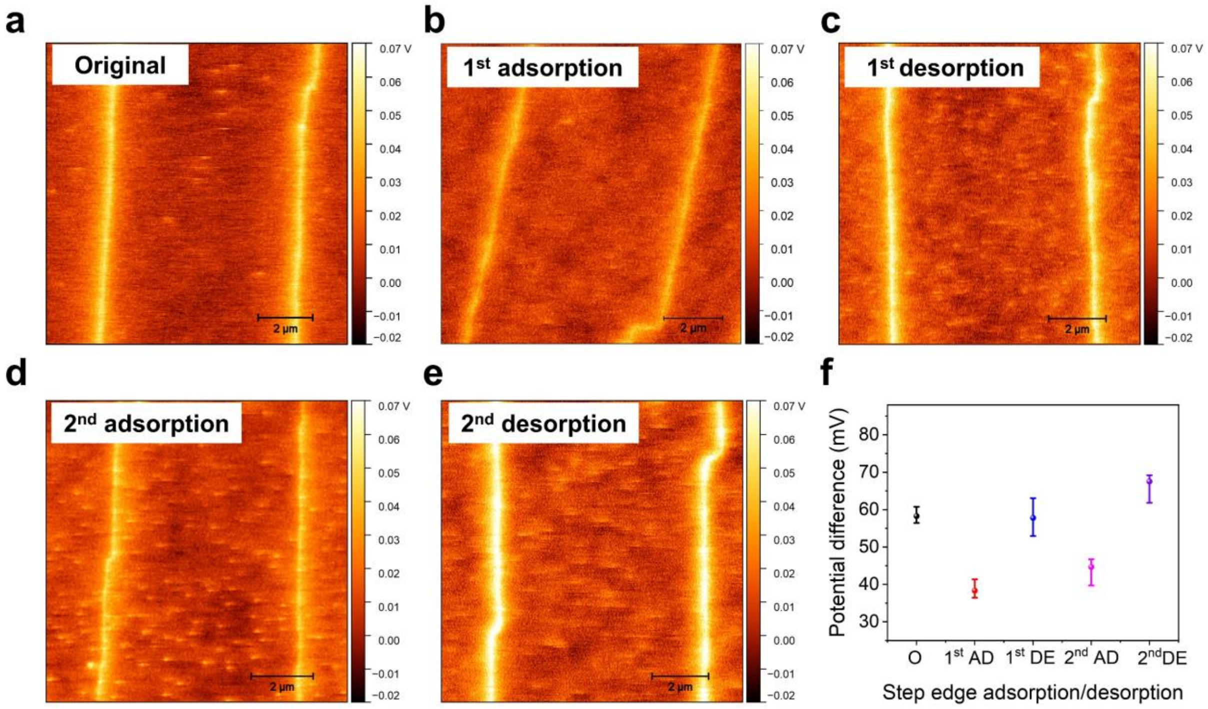

Figure S22. a-e) Corresponding KPFM surface potential images for Figure S21. f) The variations in step edge potential for the crystal exposure to NH_3 repeatedly. The reversible step edge potential suggests excellent operational stability of Cl_2-NDI single crystal sensors.

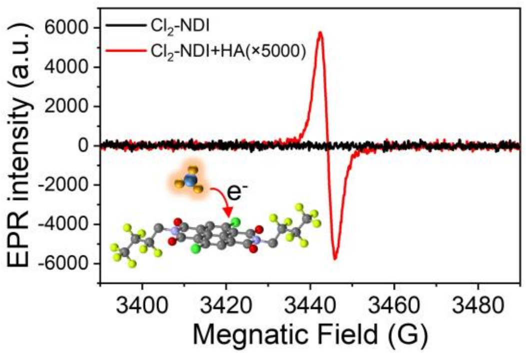

Figure S23. EPR spectrum of Cl_2-NDI (black trace) and Cl_2-NDI/hexylamine (HA, red trace) solutions. The EPR peak indicates the process of electron transfer occurring between organic amine and the Cl_2-NDI molecule.

Table S1. The comparison of the total energy (E_{total}), adsorption energy (E_{ad}), the shortest distance between NH_3 and Cl_2-NDI molecules, and the Bader charge transfer value (ΔQ_b) for the six configurations in Figure S7.

Configuration	E_{total} (eV)	E_{ad} (eV)	d (Å)	ΔQ_b (e)
1	−345.81523697	−0.08910015	3.069	0.0093
2	−345.78883199	−0.06269517	2.923	0.0120
3	−345.85025665	−0.12411983	2.309	0.0050
4	−345.84830413	−0.12216731	2.370	0.0068
5	−345.77472051	−0.04858369	2.818	0.0122
6	−345.77474448	−0.04860766	2.884	0.0064

The maximum adsorption energy and lowest total energy are observed in Configurations 3 and 4, suggesting that the adsorption of NH_3 at the lateral side of the NDI core is the most favorable. The corresponding closest distance is 2.31 Å, which is far larger than the bond lengths of H–O (0.98 Å) and H–Cl (1.27 Å), revealing the physisorption between NH_3 molecules and Cl_2-NDI

without any chemical bonding. In contrast, the peripheral fluoroalkyl chain presents minimal capacity for NH_3 adsorption. These findings suggest there is a higher probability of charge transfer at crystal step edges compared to the flat terrace. Bader charges calculation gives there is a 0.005 electron transfer from NH_3 to Cl_2-NDI molecules, which indicates the step edge traps are gradually filled as the increase of c_{NH3}. This find is consistent with linear I– semi-log c_{NH3} response and σ_s could not reach saturation even at c_{NH3} = 1%.

Table 2. Comparison of sensing performance between earlier NH_3 sensors and this work.

Material	Conductive type	Detection range	Drive voltage	Semiconductor layer	Year
CuPc, CoPc and TPFB[1a]	p-type	350 ppb–4.5 ppm	$V_G = -60$ V<br>$V_D = -60$ V	Film	2012
PDI-HIS[4]	n-type	2 ppm–100 ppm	$V_D = -60$ V	Thin film	2012
pentacene[5]	p-type	10 ppm–100 ppm	$V_G = -40$ V<br>$V_D = -40$ V	Film	2012
DTBDT-C6[6]	p-type	10 ppm–100 ppm	$V_G = -40$ V<br>$V_D = -40$ V	Ultrathin dendritic microstripes	2013
NDI(2OD)(4tBuPh)-DTYM2[7]	n-type	10 ppm–100 ppm	$V_G = +60$ V<br>$V_D = +60$ V	Films	2014
pDPPCOOH-BT[8]	p-type	10 ppb–100 ppm	$V_G = -60$ V<br>$V_D = -60$ V	Polymer film	2016
C10-DNTT[1c]	p-type	10 ppb–10 ppm	$V_G = -80$ V<br>$V_D = -80$ V	Monolayer crystals	2017
DNTT[2]	p-type	10 ppb–10 ppm	$V_G = -10$ V<br>$V_D = -10$ V	Porous film	2017
DPP2T-TT[9]	p-type	1 ppb–100 ppm	$V_G = -10$ V<br>$V_D = -20$ V	Porous film	2017
Au/Polypyrrole[10]	p-type	1 ppm–800 ppm	/	Nanofibrous film	2020
NDI3HU-DTYM2[11]	n-type	0.1 ppb–1 ppm	$V_G = +50$ V<br>$V_D = +50$ V	Monolayer molecular crystals	2020

$MoSe_2$[12]	n-type	1 ppm–50 ppm	V_D = +5 V	Nanosheets.	2020
NDI-PF and PDI-PF[13]	n-type	200 ppb–100 ppm	V_G = +40 V	Film	2021
Functionalization of graphene[14]	p-type	0.2 ppm–50 ppm	/	Film	2022
$WS_2/W_{18}O_{49}$[15]	p-type	0.5ppm–50ppm	V_g = +5 V	Film	2022
**Cl_2-NDI**	**n-type**	**5ppb–1%**	**V_G = 0 V V_D = +2 V**	**Single crystal**	**This work**

Supporting References

[1] a) W. Huang, K. Besar, R. LeCover, A. M. Rule, P. N. Breysse, H. E. Katz, *J. Am. Chem. Soc.* **2012**, *134*, 14650; b) F. I. Bohrer, A. Sharoni, C. Colesniuc, J. Park, I. K. Schuller, A. C. Kummel, W. C. Trogler, *J. Am. Chem. Soc.* **2007**, *129*, 5640; c) B. Peng, S. Huang, Z. Zhou, P. K. L. Chan, *Adv. Funct. Mater.* **2017**, *27*, 1700999.

[2] J. Lu, D. Liu, J. Zhou, Y. Chu, Y. Chen, X. Wu, J. Huang, *Adv. Funct. Mater.* **2017**, *27*, 1700018.

[3] a) S. H. Lee, B. M. Oh, C. Y. Hong, S. K. Jung, S. H. Park, G. G. Jeon, Y. W. Kwon, S. Jang, Y. Lee, D. Kim, J. H. Kim, O. P. Kwon, *ACS Appl. Mater. Interfaces* **2019**, *11*, 35904; b) M. Wu, M. He, Q. Hu, Q. Wu, G. Sun, L. Xie, Z. Zhang, Z. Zhu, A. Zhou, *ACS Sens.* **2019**, *4*, 2763; c) S. F. Liu, A. R. Petty, G. T. Sazama, T. M. Swager, *Angew. Chem., Int. Ed.* **2015**, *54*, 6554.

[4] A. Kalita, S. Hussain, A. H. Malik, N. V. V. Subbarao, P. K. Iyer, *J. Mater. Chem. C* **2015**, *3*, 10767.

[5] J. Yu, X. Yu, L. Zhang, H. Zeng, *Sens. Actuators B* **2012**, *173*, 133.

[6] L. Li, P. Gao, M. Baumgarten, K. Müllen, N. Lu, H. Fuchs, L. Chi, *Adv. Mater.* **2013**, *25*, 3419.

[7] Y. Zang, F. Zhang, D. Huang, C. Di, Q. Meng, X. Gao, D. Zhu, *Adv. Mater.* **2014**, *26*, 2862.

[8] Y. Yang, G. Zhang, H. Luo, J. Yao, Z. Liu, D. Zhang, *ACS Appl. Mater. Interfaces* **2016**, *8*, 3635.

[9] F. Zhang, G. Qu, E. Mohammadi, J. Mei, Y. Diao, *Adv. Funct. Mater.* **2017**, *27*, 1701117.

[10] Z. Li, J. Chen, L. Chen, M. Guo, Y. Wu, Y. Wei, J. Wang, X. Wang, *ACS Appl. Mater. Interfaces* **2020**, *12*, 55056.

[11] H. Li, Y. Shi, G. Han, J. Liu, J. Zhang, C. Li, J. Liu, Y. Yi, T. Li, X. Gao, C. Di, J. Huang, Y. Che, D. Wang, W. Hu, Y. Liu, L. Jiang, *Angew. Chem., Int. Ed.* **2020**, *59*, 4380.

[12] S. Singh, J. Deb, U. Sarkar, S. Sharma, *ACS Appl. Nano Mater.* **2020**, *3*, 9375.

[13] B. M. Oh, S. H. Park, J. H. Lee, J. C. Kim, J. B. Lee, H. J. Eun, Y. S. Lee, B. E. Seo, W. Yoon, J. E. Kwon, H. Yun, S. K. Kwak, O. P. Kwon, J. H. Kim, *Adv. Funct. Mater.* **2021**, *31*, 2101981.

[14] S. Freddi, D. Perilli, L. Vaghi, M. Monti, A. Papagni, C. Di Valentin, L. Sangaletti, *ACS Nano* **2022**, *16*, 10456.

[15] M. Manoharan, K. Govindharaj, K. Muthumalai, R. Pandian, Y. Haldorai, R. T. Rajendra Kumar, *ACS Appl. Mater. Interfaces* **2023**, *15*, 4703.
